# Distinguishing nontuberculous mycobacterial lung disease from pulmonary tuberculosis using radiomics machine learning models from CT images

**DOI:** 10.3389/fmed.2026.1721949

**Published:** 2026-01-21

**Authors:** Jiaofeng Zheng, Fang Wang, Xiangxin Zeng, Weiqiang Shu, Zhiyang He, Yurui Li, Yueqin Gao, Shengxiu Lv, Xueyan Liu

**Affiliations:** 1 Department of Radiology, Chongqing Public Health Medical Center, Chongqing, China; 2 Department of Research and Development, Shanghai United Imaging Intelligence, Shanghai, China; 3 Department of Radiology, Public Health Clinical Center of Chengdu, Chengdu, China

**Keywords:** computed tomography, machine learning, non-tuberculous mycobacterial lung diseases, pulmonary tuberculosis, radiomics

## Abstract

**Objectives:**

Develop and evaluate a machine learning (ML) model based on CT radiomics for the identification of non-tuberculous mycobacterial lung disease (NTM-LD) and pulmonary tuberculosis (PTB).

**Materials and methods:**

Retrospectively, chest CT images with NTM-LD and PTB patients confirmed at Medical Center 1 between January 2019 to December 2024 were collected. The dataset was divided into a training cohort and a validation cohort in a 7:3 ratio. Additionally, patients from medical center 2 were collected for external test. A radiomics model was constructed using five machine learning algorithms: Logistic Regression (LR), Random Forest (RF), Quadratic Discriminant Analysis (QDA), YeoJohnson_LR, and YeoJohnson_LR (LDA). Receiver operating characteristic (ROC) and area under the curve (AUC) were used to evaluate the diagnostic efficacy of the five models, and the optimal prediction model was obtained. The optimal model was compared with three radiologists in the testing cohort.

**Results:**

A total of 1,512 cases were included, including 1,407 cases from Center 1 (NTM-LD: 547; PTB: 860) and 105 patients from Center 2 (NTM-LD: 32; PTB: 73). Patients in the NTM-LD group were significantly older than those in the PTB group (*p* < 0.001). There was a significant gender difference between the NTM-LD group and the PTB group (*p* = 0.005). By comparing the five models, it was found that the YeoJohnson_LR (LDA) model was the best-performing prediction model, with an accuracy of 0.8286 in the external test. The AUCs of the YeoJohnson_LR (LDA) model on the training, validation, and test cohort were 0.8421, 0.8037, and 0.8233, respectively. In comparison with radiologists, the YeoJohnson_LR (LDA) model demonstrated gains of 3.12 ~ 15.62% in sensitivity and 6.85 ~ 12.33% in specificity.

**Conclusion:**

The YeoJohnson_LR (LDA) model can be used to distinguish NTM-LD from PTB, assisting in rapid clinical diagnosis and benefiting patients with NTM-LD.

## Introduction

Pulmonary tuberculosis (PTB), an infectious disease caused by *Mycobacterium tuberculosis*, is to being the world’s leading killer from a single infectious disease with 1.25 million deaths in 2023 (1). The transmission of *Mycobacterium tuberculosis* infection among the population mainly occurs through inhalation of aerosols containing *Mycobacterium tuberculosis*. Exposure to *Mycobacterium tuberculosis* can lead to primary active pulmonary tuberculosis or asymptomatic latent tuberculosis infection (LTBI) (2).

Nontuberculous mycobacteria (NTM) refer to mycobacterial infectious diseases other than *Mycobacterium tuberculosis* complex and *Mycobacterium leprae*. NTM is a common opportunistic pathogen in the environment and is prone to occur in people with weakened immune systems (3). Unlike *Mycobacterium tuberculosis*, which has received widespread attention, NTM has received much less attention. However, an insufficient understanding of NTM may lead to an underestimation of its harmfulness. NTM are ubiquitous environmental bacteria, and people can get infected by inhaling or ingesting bacteria in soil and water (4). NTM can invade multiple organs and systems throughout the body, with the lungs being the most commonly affected and having a very high mortality (5, 6).

In recent years, the infection rate of NTM has been on the rise in some countries and regions, which has drawn widespread attention (7–10). And it is reported that the NTM separation rate in China has increased (11–13). An analysis of sputum samples from suspected cases collected at 72 national tuberculosis surveillance sites in 31 provinces of the Chinese mainland revealed that among 4,917 mycobacterial isolates, 6.4% were NTM (11). Environmental pollution, population aging, and the use of immunosuppressants may have contributed to the infection rate of NTM. Although technological development and medical progress have to some extent increased the detection rate of NTM, the diagnosis of NTM lung diseases (NTM-LD) remains challenging due to the similar clinical and imaging manifestations of NTM-LD and PTB. The existing diagnostic methods mainly rely on etiological detection and CT examination, but they have certain limitations (14, 15). To explore the potential characteristic differences between NTM-LD and PTB, it is urgent to develop a new, efficient, sensitive, and economical technical means to assist in differential diagnosis.

Recently, radiomics has been increasingly widely applied in the medical field. By mining data features, it has shown great potential in assisting the diagnosis (16, 17), differentiation (18, 19), treatment (20, 21) and prediction (22, 23) of diseases. This provides new inspiration for identifying NTM-LD from PTB.

This study aims to construct radiomics models of NTM-LD and PTB using five machine learning algorithms (Z_score_LR, Z_score _RF, Z_score _QDA, YeoJohnson_LR, YeoJohnson_LR (LDA)). To explore the value of computed tomography (CT) radiomics in the differentiation of NTM-LD and PTB.

## Materials and methods

### Patients and dataset

**Figure 1:**
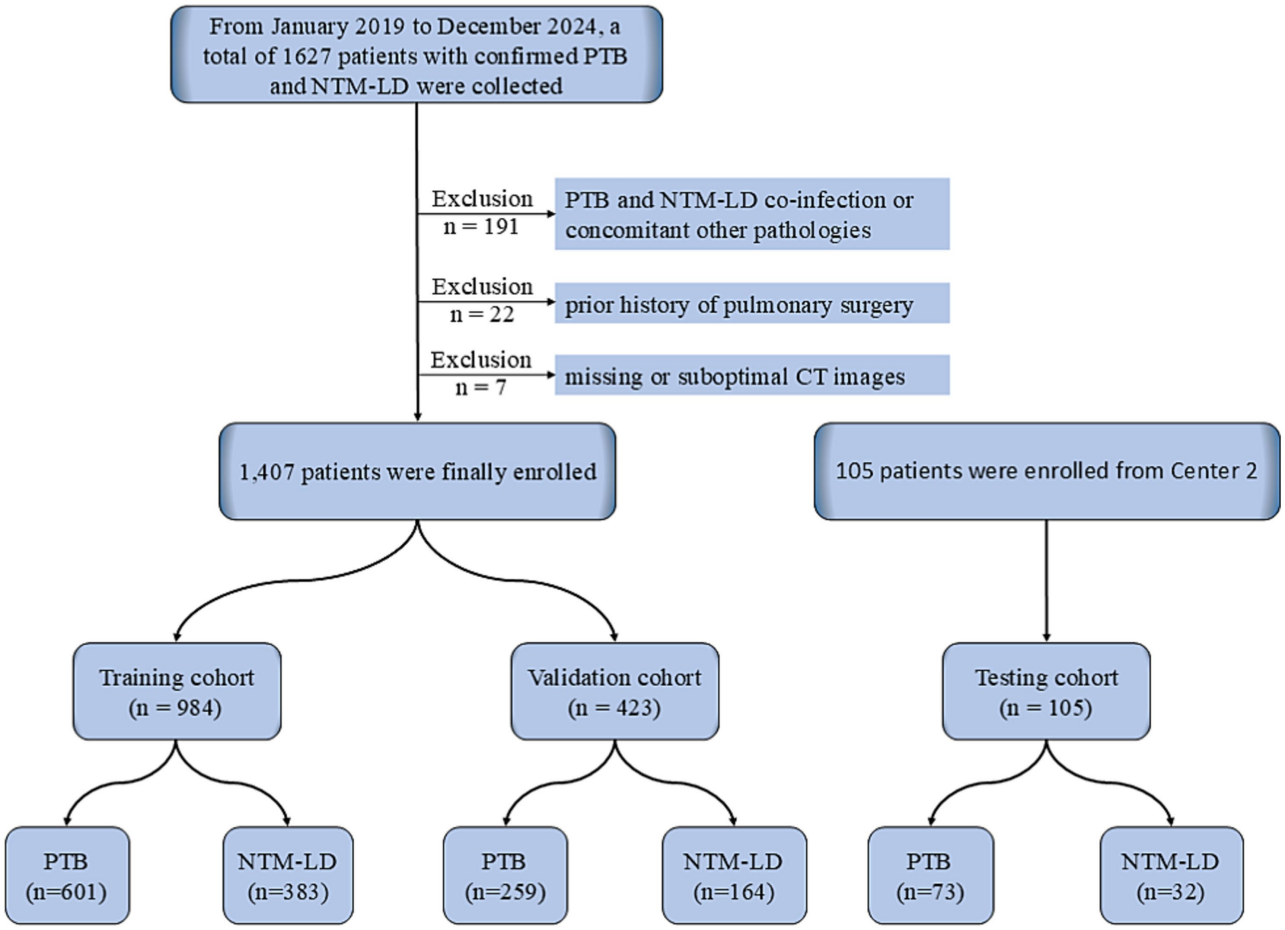
Flowchart of patient screening. PTB, pulmonary tuberculosis; NTM-LD, nontuberculous mycobacteria lung disease.

This retrospective study received ethical approval from the Ethics Committee of Chongqing Public Health Medical Center (Approval No. 2024-019-02-KY, Center 1) and was granted an informed consent waiver for the period spanning January 2019 to December 2024. Definitive diagnosis was established through microbiological confirmation according to established reference standards: NTM-LD infections were diagnosed following *the Treatment of Nontuberculous Mycobacterial Pulmonary Disease: An Official ATS/ERS/ESCMID/IDSA Clinical Practice Guidelines (version 2020)* (24), while PTB diagnoses adhered to the *National Health Commission of the People’s Republic of China. In: Diagnostic criteria. For pulmonary tuberculosis (WS 288-2017)* (25). Inclusion criteria comprised: (1) age ≥ 18 years; (2) microbiologically confirmed PTB or NTM-LD; and (3) availability of pretreatment CT images. Exclusion criteria encompassed: (1) co-infection with both TB and NTM-LD; (2) concomitant pulmonary pathologies, including lung cancer, fungal infections, or pneumoconiosis, etc.; (3) prior history of pulmonary surgery; and (4) missing or suboptimal CT images precluding reliable analysis. Identical ethical and diagnostic criteria were employed for data collection at Center 2. Baseline demographic characteristics (age and sex) were documented for all enrolled patients, with non-contrast CT scans acquired within 30 days before treatment initiation. Patient screening and enrollment was shown in Figure 1.

### CT image acquisition

CT image acquisition was performed using two scanner models: the GE Optima CT680 Expert 64-slice CT scanner (Center 1) and the GE BrightSpeed CT scanner (Center 2). All patients were positioned supine and scanned during a single breath-hold at full inspiration, covering the entire lung volume from the apex to the costophrenic angles. Standard non-contrast scans were acquired with a tube voltage of 120 kV and automated tube current modulation. Images were reconstructed with a slice thickness and interval of 5 mm using a 512 × 512 matrix. For the Optima CT680 Expert scanner (Center 1), the in-plane pixel resolution ranged from 0.312 mm × 0.312 mm to 0.977 mm × 0.977 mm. For the BrightSpeed scanner (Center 2), spatial resolution was 20 lp/cm with a density resolution of 0.3%. To standardize images for subsequent radiomics analysis and minimize variations due to differences in slice thickness or other acquisition parameters between scans, the CT values (Hounsfield Units, HU) from each scan were interpolated to a voxel size of 1 mm × 1 mm × 5 mm using a lung window setting (window level/width: -600 HU / 1,500 HU).

### Lungs segmentation and radiomics feature extraction

All images were processed using the uAI Research Portal (Version: 20250130sp1), an independently developed research platform by Shanghai United Imaging Intelligent Medical Technology Co., Ltd. Automatic segmentation of bilateral lung fields was performed using the VB-Net deep learning model. This model demonstrated high performance in our prior work, achieving a mean Dice similarity coefficient (DSC) of 0.989 ± 0.004 (mean ± SD), and has been validated in other pulmonary infection studies (26, 27). Following automated segmentation, all results underwent expert review by board-certified thoracic radiologists (>10 years’ experience), with any inaccuracies manually corrected.

Radiomics feature extraction from the segmented lung volumes was implemented using PyRadiomics (version 3.8.8). Image preprocessing included resampling to an isotropic in-plane resolution of 0.7 × 0.7 × 5 mm^3^ and gray-level discretization into 25 fixed bins using nearest-neighbor interpolation. A total of 2,264 radiomics features were extracted, including 450 first-order features, 14 shape features, 525 gray level co-occurrence matrix (GLCM) features, 350 gray level dependence matrix (GLDM) features, 400 gray level run length matrix (GLRLM) features, 400 gray level size zone matrix (GLSZM) features, and 125 neighboring gray tone difference matrix (NGTDM) features.

### Radiomics feature selection and radiomics model construction

Radiomics feature selection was performed using a sequential approach: Pearson correlation analysis (*p* < 0.05) identified relevant features, followed by Least Absolute Shrinkage and Selection Operator (LASSO) regression with 10-fold cross-validation to refine the most predictive subset, retaining only features with non-zero coefficients. The optimal regularization penalty (*λ*) was determined based on the criterion of minimum binomial deviance plus one standard deviation. A patient-specific radscore was subsequently computed for each subject using the linear predictor derived from the LASSO model.

**Figure 2:**
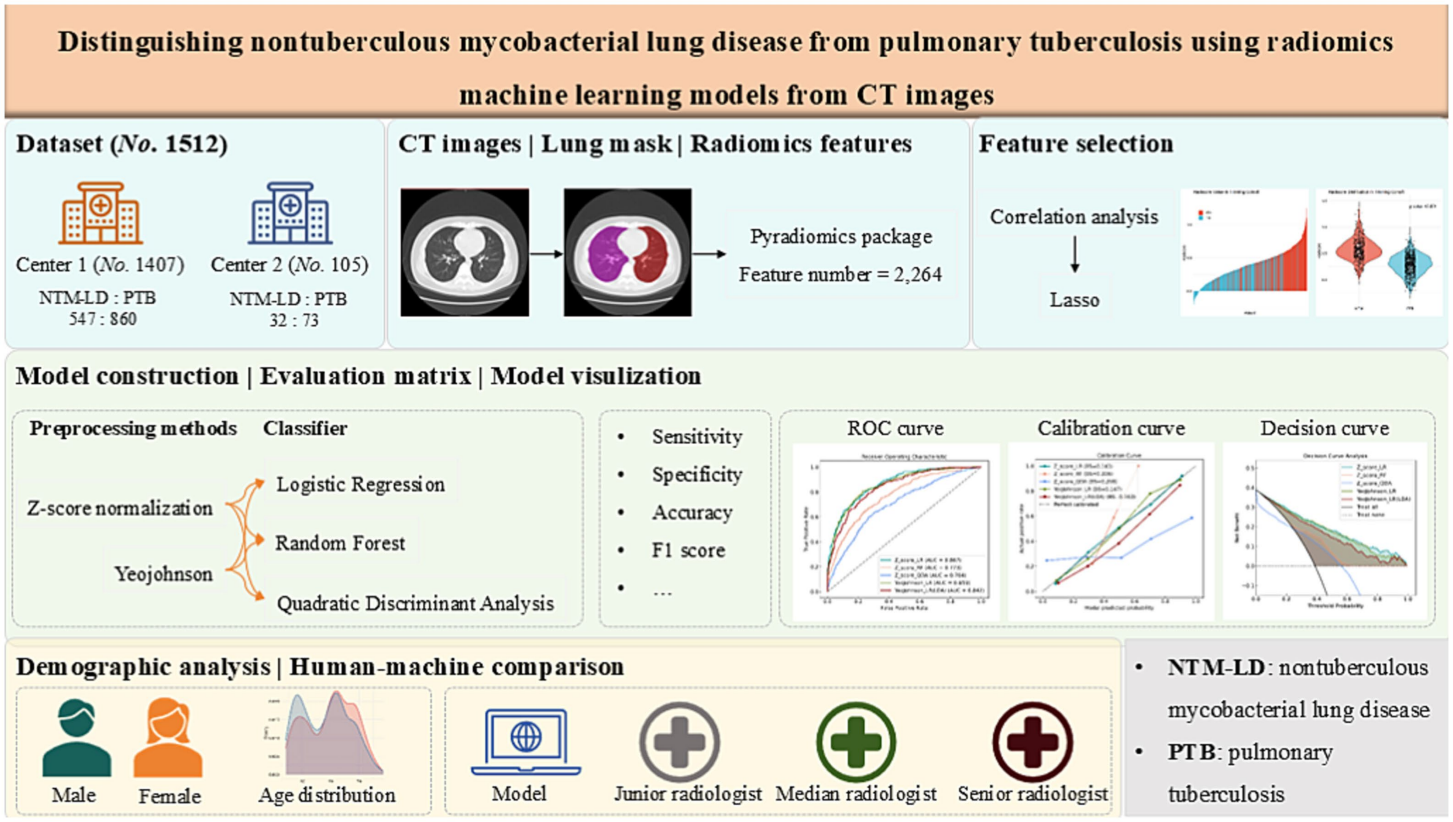
The entire workflow of model development, validation, testing, and comparison.

Using the selected features, predictive models were trained on the training cohort employing three distinct MLalgorithms: LR, RF, and QDA. We systematically evaluated the impact of feature normalization strategies—comparing Z-score normalization against Yeo-Johnson transformation—and examined the influence of dimensionality reduction by constructing models both with and without prior application of Linear Discriminant Analysis (LDA). Finally, we have constructed five models, including Z_score_LR, Z_score _RF, Z_score_QDA, YeoJohnson_LR and YeoJohnson_LR (LDA). Hyperparameter tuning and model selection were performed using the validation cohort. The final performance and generalizability of the optimal model were rigorously assessed on an independent testing cohort from Center 2. The step-by-step workflow for radiomics model development and validation is provided in Supplementary material 1. And the entire workflow of model development, validation, testing, and comparison was illustrated in Figure 2.

### Statistical analysis

Statistical analyses were performed using R software (version 4.4.1; R Foundation for Statistical Computing, Vienna, Austria). Model discrimination was evaluated using receiver operating ROC and AUC. Calibration was assessed with the Hosmer-Lemeshow test and calibration curves, while clinical utility was quantified via decision curve analysis (DCA). To compare model performance, the DeLong test analyzed differences in AUC values, and the net reclassification index (NRI) and integrated discrimination improvement (IDI) measured the incremental predictive value of the optimal model over compared methods. Additionally, three radiologists independently reviewed CT images in the testing cohort under blinded conditions to differentiate NTM-LD from PTB, with the understanding that each patient had a single-pathogen infection. Performance metrics—including AUC, accuracy, sensitivity, specificity, precision, and F1 score—were calculated for both the machine learning models and radiologists. Confusion matrices provided visual comparisons of their diagnostic outcomes. Statistical significance was defined as *p* < 0.05, with *p*-values adjusted for multiple testing using Benjamini-Hochberg method where applicable.

## Results

### Baseline characteristics

**Figure 3:**
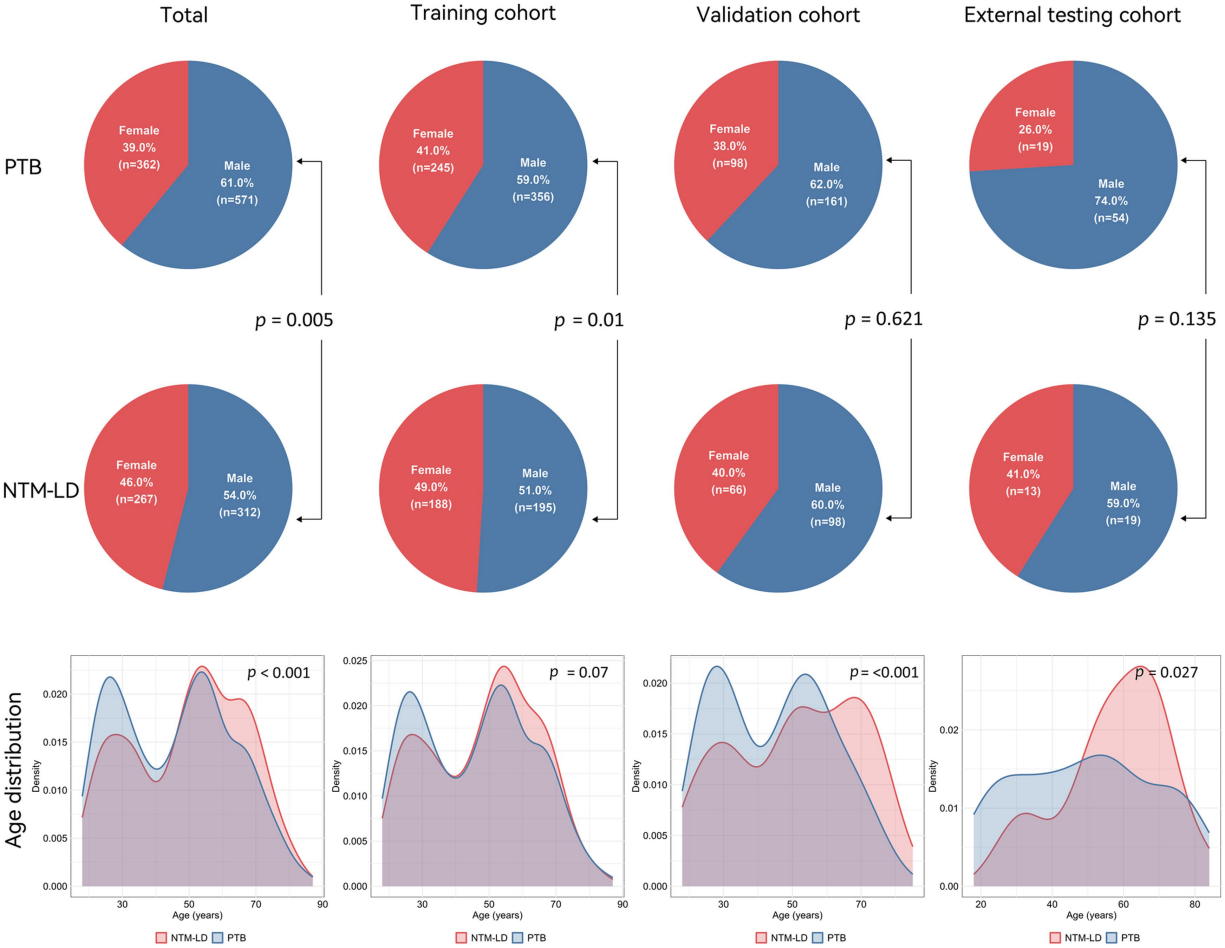
Pie charts of demographic analysis (top row: PTB, middle row: NTM-LD) and density plots of age distribution (bottom row) between NTM-LD and PTB patients across total, training, validation, and testing cohort. *p* < 0.05 were considered statistically significant.

This study included 1,407 cases from Center 1, comprising 547 patients with NTM-LD (mean age:50 ± 18 years; 293 male) and 860 patients with PTB (mean age:46 ± 17 years; 517 male). Data were randomly split into a training cohort (70%, *n* = 984) and a validation cohort (30%, *n* = 423) at a 7:3 ratio. Additionally, 105 patients from Center 2 were collected for external testing, including 32 NTM-LD (mean age:57 ± 15 years; 19 male) and 73 PTB (mean age:48 ± 20 years; 54 male) patients. Patients in the NTM-LD group were significantly older than those in the PTB group (*p* < 0.001). There was a significant gender difference between the NTM-LD group and the PTB group (*p* = 0.005). Baseline characteristics were detailed in Table 1 and Figure 3.

**Table 1:** Patient baseline characteristics in two centers.

Characteristics	PTB	NTM-LD	*p* value
Total patients (*N*)	933	579	–
Age (year, quantile)	48 [29, 58]	53 [34, 65]	<0.001
Gender/Male (*N*, %)	571 (61)	312 (54)	0.005
Training cohort (*N*)	601	383	–
Age (year, quantile)	48 [29, 58]	52 [34, 63]	0.070
Gender/ Male (*N*, %)	356 (59)	195 (51)	0.010
Validation cohort (*N*)	259	164	–
Age (year, quantile)	47 [30, 58]	53 [33, 67]	<0.001
Gender/ Male (*N*, %)	161 (62)	98 (60)	0.621
External testing cohort (*N*)	73	32	–
Age (year, quantile)	49 [30, 62]	60 [51, 69]	0.027
Gender/ Male (*N*, %)	54 (74)	19 (59)	0.135

Mann–Whitney U test was used for age because of non-normal distribution and chi-square test was used for gender. Statistical analysis was performed with python 3.10.6. All statistical tests were two-sided, and *p* value lower than 0.05 were considered statistically significant.

### Feature selection

**Figure 4:**
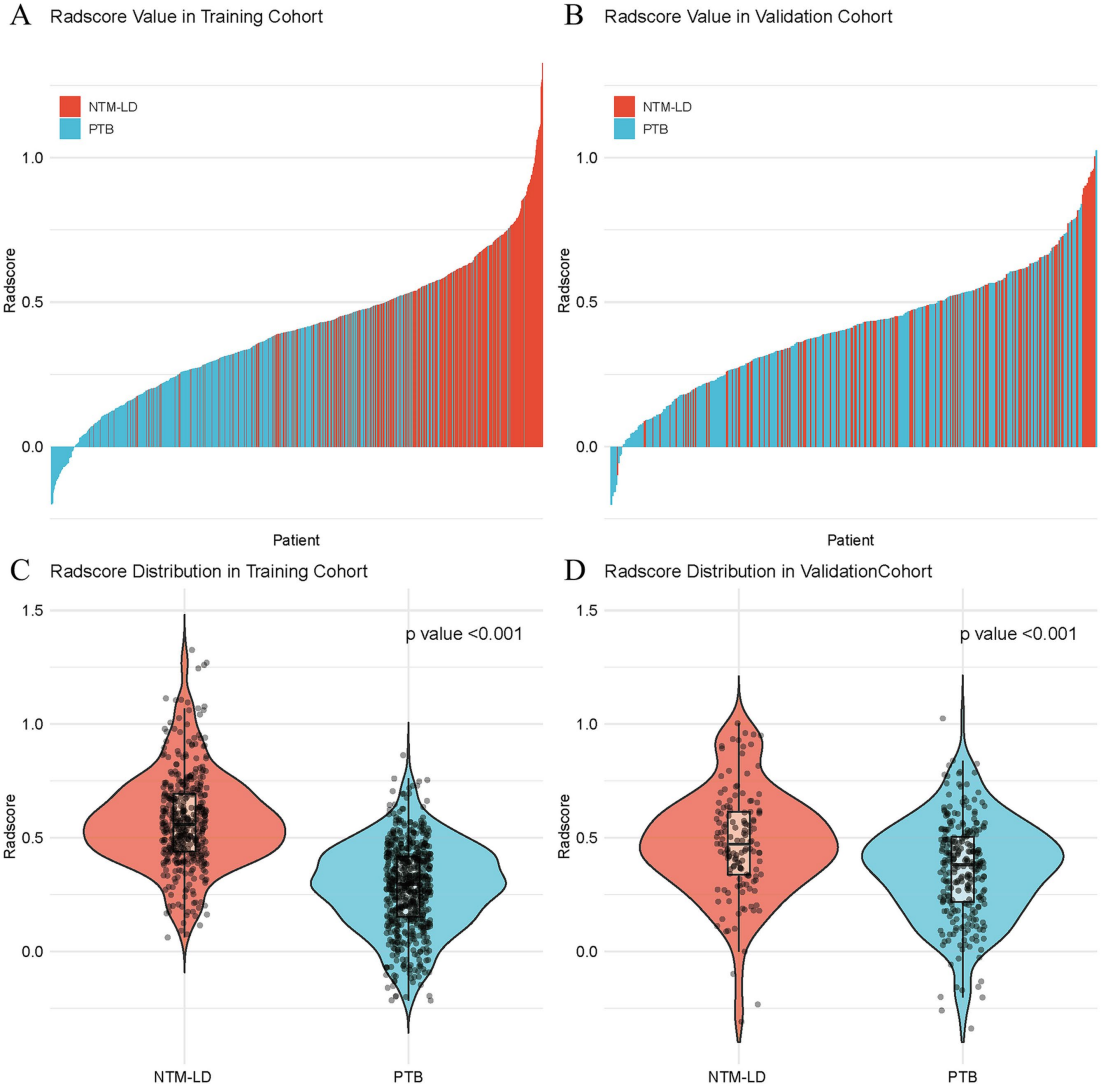
Radscore distributions between NTM-LD and PTB patients in the training cohort and the validation cohort. The top panel displays rank-ordered Radscore profiles for individual patients in the training **(A)** and validation **(B)** cohorts. The bottom panel presents violin plots of Radscore distributions, where the shape of the violins reflects data density in the training **(C)** and validation **(D)** cohorts. In both the training and validation cohorts, NTM-LD and PTB exhibit significantly different Radscore distributions (*p* < 0.001). PTB, pulmonary tuberculosis; NTM-LD, nontuberculous mycobacteria lung disease.

A total of 2,264 radiomics features were extracted from non-contrast CT scans. Following selection of 1,414 features based on Pearson correlation (*p* < 0.05), LASSO regression with 10-fold cross-validation identified 85 robust features. Among these the three most influential features for the model were glszm_wavelet.LLL.ZonePercentage (ZP), gldm_log.sigma.2.0.mm.3D.DependenceVariance (DV), and glszm_log.sigma.1.0.mm.3D.GrayLevelVariance (GLV), with LASSO coefficients of 0.104, 0.059, and 0.058, respectively (see Supplementary material 2; Table 1). And the top 15 discriminative features differentiating NTM-LD from PTB groups detailed in Table 2 of Supplementary material 2. Notably, the Radscore derived from these features demonstrated significantly higher values in NTM-LD patients compared to PTB patients across both training and validation cohorts (*p* < 0.001), as visualized in Figure 4.

**Table 2:** Performance of various models for predicting NTM-LD.

Models	Group	AUC (95% CI)	Sensitivity	Specificity	Accuracy	Precision	F1_Score
Z_score_LR	Training	0.8673 (0.8452–0.8673)	0.7990	0.7587	0.7744	0.7587	0.7783
	Validation	0.7411 (0.6931–0.7411)	0.7195	0.6448	0.6738	0.6448	0.6801
	Testing	0.7526 (0.6543–0.7526)	0.6176	0.7368	0.6977	0.7368	0.6720
Z_score _RF	Training	0.7732 (0.7438–0.7732)	0.6475	0.7587	0.7154	0.7587	0.6987
	Validation	0.6996 (0.6503–0.6996)	0.5549	0.6718	0.6241	0.6680	0.6062
	Testing	0.7276 (0.6311–0.7276)	0.5882	0.7263	0.6899	0.7263	0.6500
Z_score _QDA	Training	0.7040 (0.6708–0.7040)	0.6005	0.7321	0.6809	0.7321	0.6598
	Validation	0.6725 (0.6211–0.6725)	0.5549	0.6718	0.6265	0.6718	0.6078
	Testing	0.6211 (0.5173–0.6211)	0.4706	0.7684	0.6822	0.7684	0.5837
YeoJohnson _LR	Training	0.8594 (0.8364–0.8594)	0.8068	0.7704	0.7846	0.7704	0.7882
	Validation	0.7413 (0.6935–0.7413)	0.7012	0.7223	0.7141	0.6149	0.6558
	Testing	0.7641 (0.6726–0.7641)	0.7064	0.7162	0.7134	0.4715	0.5650
YeoJohnson_LR (LDA)	Training	0.8421 (0.8176–0.8421)	0.7885	0.7354	0.7561	0.7354	0.7611
	Validation	0.8037 (0.7615–0.8037)	0.7866	0.6757	0.7187	0.6757	0.7269
	Testing	0.8233 (0.7242–0.9224)	0.6875	0.8904	0.8286	0.7333	0.7097

LR, Logistic Regression; RF, Random Forest; QDA, Quadratic Discriminant Analysis; LDA, Linear Discriminant Analysis; Z_score, Z score normalization; YeoJohnson, YeoJohnson transformer.

### Performance of radiomics models in NTM-LD versus PTB classification

**Figure 5:**
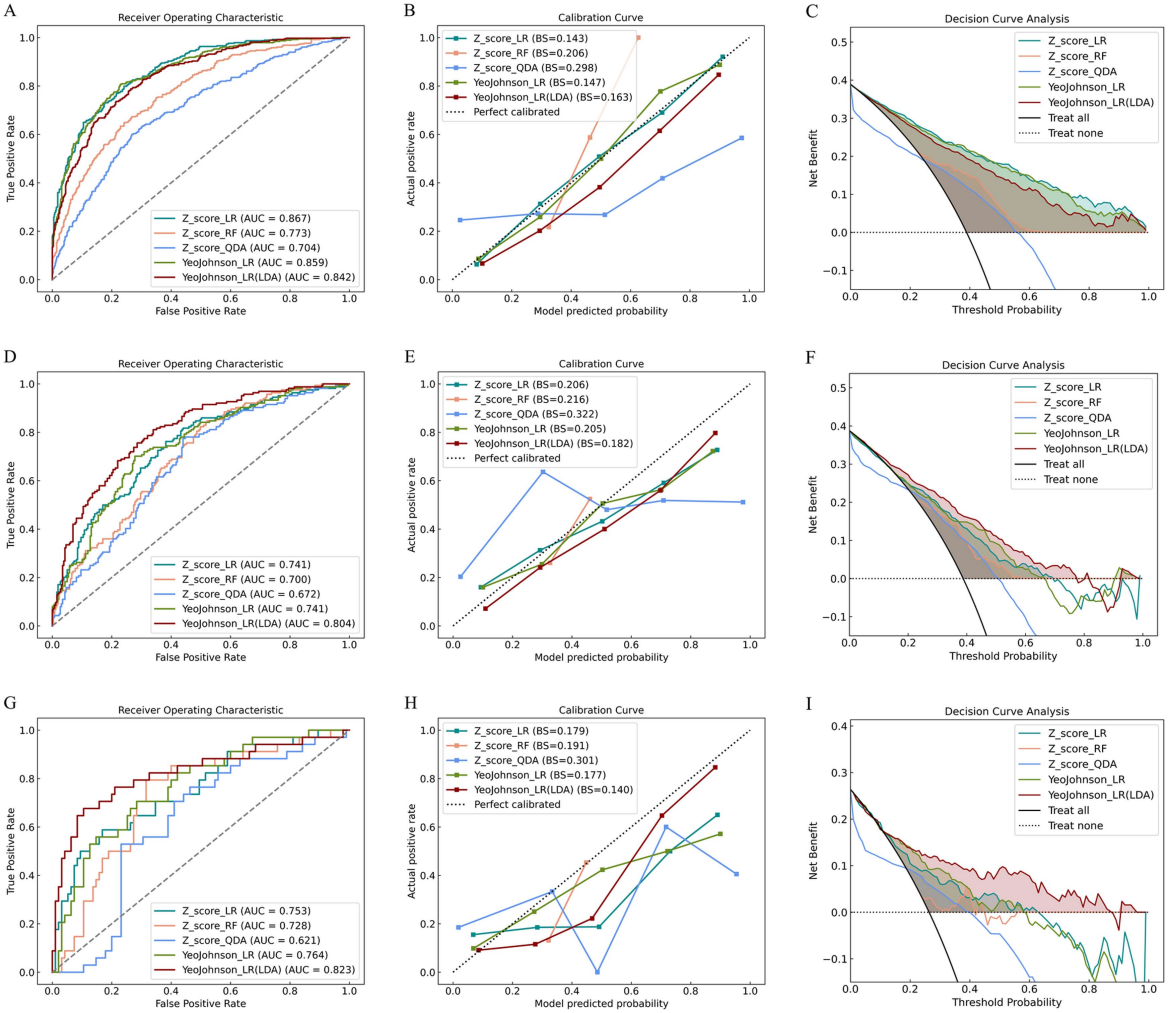
Performance evaluation of the model across training, validation, and testing cohorts. The figure presents ROC curves **(A,D,G)**, calibration curves **(B,E,H)**, and clinical decision curves **(C,F,I)** for the training cohort (top row), validation cohort (middle row), and testing cohort (bottom row), respectively. Each row consistently displays the three evaluation metrics for its respective cohort, demonstrating the model’s diagnostic performance, prediction accuracy, and clinical utility across development and independent validation stages.

The machine learning models exhibited varying performance depending on normalization strategies and algorithms. Specifically, under Z-score normalization, Z_score_LR outperformed Z_score _RF and Z_score _QDA in the testing cohort, with AUC of 0.7526 vs. 0.7276 (RF, *p* = 0.5745) and 0.6211 (QDA, *p* = 0.0166), sensitivity of 0.6176 vs. 0.5882 (RF, *p* = 0.7539) and 0.4706 (QDA, *p* < 0.001), and accuracy of 0.6977 vs. 0.6899 (RF, *p* = 0.5114) and 0.6822 (QDA, *p* = 0.5032). Building on this baseline, YeoJohnson normalization further improved Z_score_LR performance, yielding a + 1.15% AUC gain (0.7641, *p* = 0.5966) and +1.57% accuracy improvement (0.7134, *p* = 0.0215). Crucially, the incorporation of LDA prior to the LR classifier significantly enhanced model performance, achieving a state-of-the-art AUC of 0.8233 (+7.07% vs. baseline Z_score_LR, *p* = 0.0061; +5.92% vs. YeoJohnson_LR, *p* = 0.0094). Beyond discriminative metrics, calibration analysis revealed YeoJohnson_LR (LDA) most closely approximated the ideal calibration line, with Brier scores of 0.182 (validation) and 0.140 (testing). And decision curve analysis further demonstrated YeoJohnson_LR (LDA)'s consistent superiority across the clinically relevant threshold spectrum (0.15–1.0), yielding higher net benefit than all comparator models. Significant reclassification improvement was confirmed by Delong test (*p* = 0.0094), NRI (0.1703, *p* = 0.0129) and IDI (0.0851, *p* = 0.0003) versus YeoJohnson_LR. Tables 2, 3 detail the individual model performance and provide a comparative analysis in training, validation, and testing cohorts. And Figure 5 provided various line charts to visualize the models’ evaluation performance and clinical net benefit.

**Table 3:** Statistical comparison of model performance.

Group	Compared method	Delong test		NRI test			IDI test		
		AUC	AUC	NRI score	NRI	NRI	IDI score	IDI	IDI
		Z value	*p* value		Z value	*p* value		Z value	*p* value
Training	Z_score_LR vs. YeoJohnson_LR	1.5122	0.1305	−0.009	−0.471	0.6376	−0.0214	−3.6393	0.0003
	Z_score_LR vs. YeoJohnson_LR (LDA)	3.6389	0.0003	−0.0251	−1.0424	0.2972	−0.0379	−4.4152	<0.001
	Z_score_RF vs. YeoJohnson_LR (LDA)	5.0524	<0.001	0.3201	9.7492	<0.001	0.269	19.8409	<0.001
	Z_score_QDA vs. YeoJohnson_LR (LDA)	9.1464	<0.001	0.1983	5.8641	<0.001	0.0489	1.9992	0.0456
	YeoJohnson_LR vs. YeoJohnson_LR (LDA)	3.0233	0.0025	−0.0161	−0.7504	0.453	−0.0166	−2.4342	0.0149
Validation	Z_score_LR vs. YeoJohnson_LR	0.0185	0.9853	0.0764	2.3277	0.0199	−0.0083	−0.7032	0.4819
	Z_score_LR vs. YeoJohnson_LR (LDA)	3.922	0.0001	0.1543	3.4735	0.0005	0.0545	3.3131	0.0009
	Z_score_RF vs. YeoJohnson_LR (LDA)	4.2025	<0.001	0.3255	6.2946	<0.001	0.2354	11.1775	<0.001
	Z_score_QDA vs. YeoJohnson_LR (LDA)	4.7921	<0.001	0.2073	3.6509	0.0003	0.0401	0.9917	0.3214
	YeoJohnson_LR vs. YeoJohnson_LR (LDA)	5.2247	<0.001	0.0779	2.0372	0.0416	0.0628	5.2849	<0.001
Testing	Z_score_LR vs. YeoJohnson_LR	0.5966	0.5508	0.0000	0.0000	1.000	−0.0253	−1.0622	0.2881
	Z_score_LR vs. YeoJohnson_LR (LDA)	2.7432	0.0061	0.1703	2.4875	0.0129	0.0598	1.9461	0.0516
	Z_score_RF vs. YeoJohnson_LR (LDA)	2.2999	0.0215	0.5146	5.1415	<0.001	0.3051	6.2598	<0.001
	Z_score_QDA vs. YeoJohnson_LR (LDA)	4.0249	0.0001	0.2839	2.9126	0.0036	0.1575	1.8995	0.0575
	YeoJohnson_LR vs. YeoJohnson_LR (LDA)	2.5987	0.0094	0.1703	2.4875	0.0129	0.0851	3.5957	0.0003

NRI, Net Reclassification Index; IDI, Integrated Discrimination Improvement. All *p* values adjusted for multiple testing using the Benjamini-Hochberg procedure.

### Comparison of performance between optimal model and radiologists

**Figure 6:**
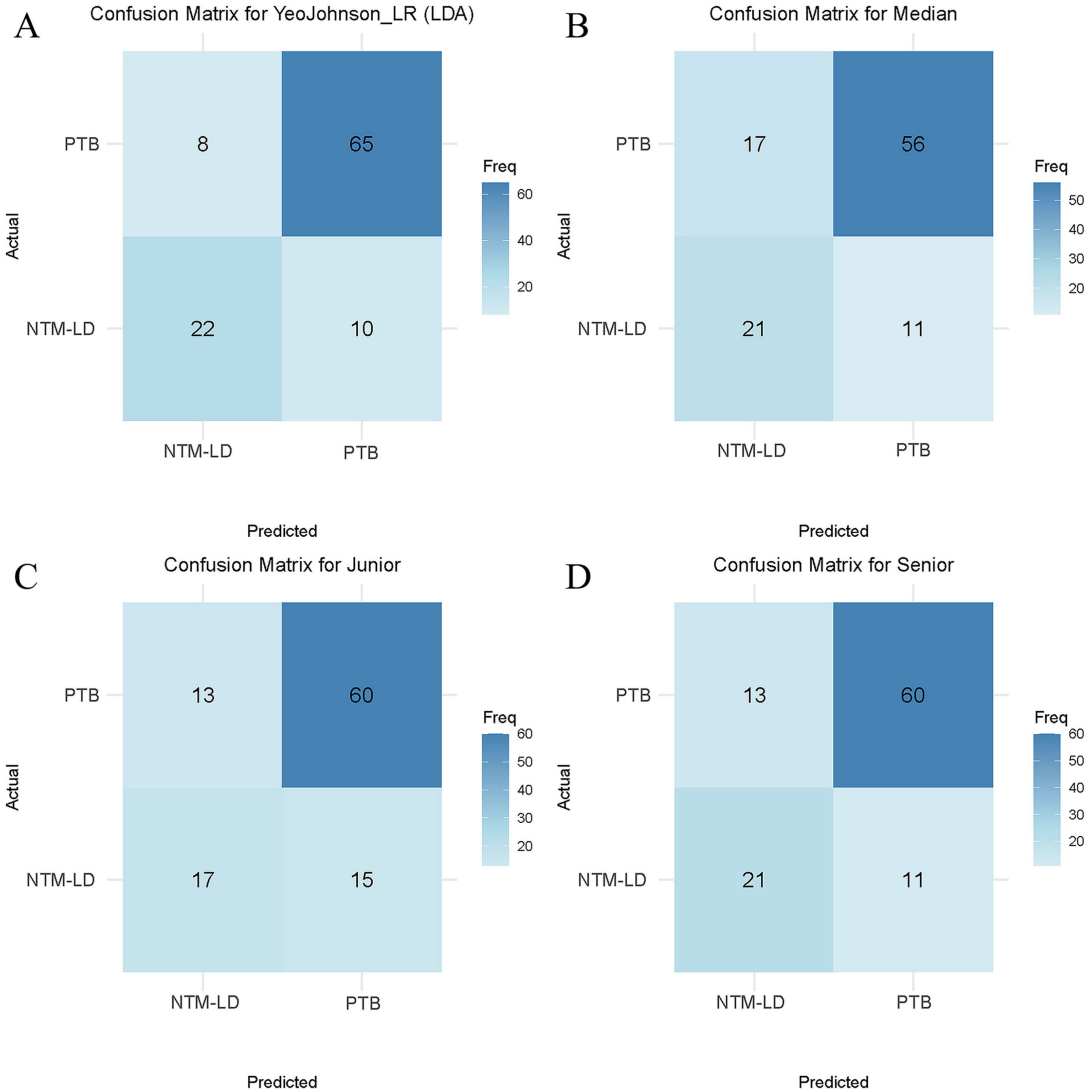
Diagnostic performance comparison. Confusion matrices for the optimal radiomics model (YeoJohnson_LR (LDA), **A**) and three radiologists: junior **(C)**, median **(B)**, and senior **(D)**. Color intensity corresponds to prediction frequency (darker blue = higher counts).

The YeoJohnson_LR (LDA) model demonstrated superior diagnostic performance compared to all three radiologists, regardless of experience level (Table 4). Its significant advantage is quantified across key metrics: achieving a substantially higher AUC (0.8233) than the radiologists (ranging from 0.6773 to 0.7391, representing improvements of 8.42 to 14.60%). Similarly, the model’s accuracy (0.8286) surpassed that of the junior and median-experience radiologists by 9.53% (both 0.7333) and the senior radiologist by 5.72% (0.7714). Notably, the model exhibited its largest performance gap in specificity (0.8904), outperforming the radiologists by 6.85 to 12.33%, highlighting a particular strength in reducing false positives. It also showed higher sensitivity (0.6875 vs. 0.5313 ~ 0.6563, +3.12% to +15.62%), significant gains in precision (+11.57% ~ 16.66%) and F1 score (+7.33% ~ 16.13%). These results collectively demonstrate the model’s consistent superiority in diagnostic accuracy, particularly its ability to minimize false-positive rates. The human-machine comparison was detailed in Table 4, Figure 6 represents the confusion matrix of YeoJohnson_LR (LDA) and three radiologists.

**Table 4:** Comparison of effectiveness between the YeoJohnson_LR (LDA) model and radiologists.

Model	AUC (95% CI)	Accuracy	Sensitivity	Specificity	Precision	F1Score
YeoJohnson_LR (LDA)	**0.8233 (0.7242–0.9224)**	**0.8286**	**0.6875**	**0.8904**	**0.7333**	**0.7097**
Junior	0.6773 (0.5780–0.7756)	0.7333	0.5313	0.8219	0.5667	0.5484
Median	0.7122 (0.6152–0.8086)	0.7333	0.6563	0.7671	0.5526	0.6000
Senior	0.7391 (0.6453–0.8345)	0.7714	0.6563	0.8219	0.6176	0.6364

Junior, Median, and Senior denote radiologists with 5, 10, and 15 years of clinical experience, respectively. Bold values represent the highest performance.

## Discussion

In this study, we included baseline characteristics such as gender and age. We found that patients in the NTM-LD group were significantly older than those in the PTB group (*p* < 0.001), which was consistent with the results of previous studies (15, 28–30). The elderly are prone to NTM-LD infection. This might be related to the fact that NTM is an opportunistic pathogen. The immunity of the elderly may decline as they get older, making them more prone to illness (12, 29). Our study revealed that NTM-LD is more prevalent in females (*p* = 0.005). It has been reported that NTM-LD is more common among older women; specifically, morbidity and disease risk increase markedly in women aged ≥65 years (8, 31). However, some other studies have found that the incidence of NTM-LD and PTB has no relation to gender (32–34). Males and females differ in pathogenic infections due to the sexes providing different genetic backgrounds, anatomic niches, immunological profiles, and hormonal environments that can directly affect pathogens as well as the development of diseases following infection (35). Thus, the baseline characteristics of the patients alone are not sufficient to distinguish between NTM-LD and PTB.

Regarding clinical symptoms, both NTM-LD and PTB may present with symptoms such as cough, fever, dyspnea, night sweats, fatigue, weight loss, etc. In the study by Kim C et al. (33), dyspnea was more common in patients with PTB, while cough was more prevalent in NTM-LD patients (*p* < 0.001). Contrary to the results of Kim et al., in the study by Wangle L et al. (15), cough was more common in PTB, while dyspnea was more common in NTM-LD. Giller D et al. (36) and Klann E et al. (37) found that weakness, dyspnea, fever, weight loss, and night sweats were more common in PTB, while hemoptysis and pulmonary hemorrhage were more common in NTM-LD (*p* < 0.05) (38). And Zhang W et al. (29) revealed symptoms such as cough, sputum production, fever, dyspnea, shortness of breath, hemoptysis, chest pain, fatigue, emaciation, and appetite have no significant difference between NTM-LD and PTB. Therefore, it is impossible to distinguish patients with NTM-LD from those with PTB solely based on clinical symptoms.

Bacterial culture and strain identification are the gold standard for diagnosing NTM-LD. However, these methods are difficult to be widely applied due to their time-consuming (2 ~ 6 weeks) nature (14, 39), economic factors, and the requirements of laboratory conditions. Several commonly used laboratory methods (such as AFB, Xpert, and T-SPOT) also have certain limitations. Acid-fast bacilli (AFB) sputum smear is widely used and the most efficient procedure for the initial screening of PTB. The presence of AFB in the stained sputum (AFB smear-positive) indicates a preliminary diagnosis of pulmonary mycobacterial infection (40). However, a positive AFB smear is not specific for PTB; both PTB and NTM-LD show positive results (41, 42). T-SPOT is an interferon-*γ* release assay (IGRA) used to detect tuberculosis. And NTM infection may also result in a positive T-SPOT result (14). Xpert is a molecular testing method used for detecting tuberculosis, but its results may have the possibility of false negatives (41). Notedly, Xpert and T-SPOT not only require professional equipment and technical support but also cannot distinguish between active tuberculosis and LTBI. In countries with a high burden of tuberculosis, a considerable proportion of the population suffers from LTBI (43), which may lead to positive results in Xpert or T-SPOT tests. So, laboratory tests have limitations in enabling early diagnosis. In many tuberculosis epidemic areas, it is not uncommon for patients to receive empirical anti-tuberculosis treatment while waiting for the results of the bacterial culture (15). This may lead to the burden of adverse drug reactions and economic costs.

CT examination has the advantages of being non-invasive, available, and efficient, and is widely used in the screening, diagnosis, and follow-up of lung diseases (44–46). Although the pulmonary manifestations of NTM-LD and PTB are highly similar (40, 47), there are still some differences. For instance, Yuan M et al. (40) discovered that the prevalence of pleural effusion, nodules < 10 mm in size, tree-in-bud pattern, and bronchiectasis in PTB patients was significantly higher than NTM-LD patients (*p* < 0.05). The Chu HQ et al. (42) compared the imaging examinations of two groups of patients and found that the NTM-LD patients more frequently had bronchiectasis, thin-walled cavity (D ≥ 3 cm) (*p* < 0.05), and the right middle lobe and left lingualar segment bronchiectasis were more prominent in NTM-LD (*p* < 0.001). Noted, bronchiectasis in the right middle lobe and left lingualar segment is regarded as a key characteristic of NTM-LD (42, 48). The thin-walled cavity is also considered to be more indicative of NTM-LD rather than PTB (33). Certain imaging differences between NTM-LD and PTB enable machine models based on imaging data to distinguish between them. So far, there are very few studies on radiomics in differentiating NTM-LD from PTB. As far as we know, only a few reports have been published (30, 34, 47, 49, 50).

In this study, we developed and evaluated a set of machine learning models based on radiomics for differentiating NTM-LD from PTB. The results showed that the YeoJohnson_LR (LDA) model had the best diagnostic efficacy. In this study, we selected LR, RF, and QDA classifiers. The results showed that the LR classifier was the best-performing predictive model. Similar to the reports in the literature, previous studies have also shown that the LR model has a good recognition effect in differentiating NTM-LD from PTB. For example, Li HL et al. ^[30]^constructed a radiomics model for discriminating NTM-LD from PTB, and found that the LRmodel was the optimal model. Its AUC, sensitivity, and specificity in the external test were 0.766, 0.833, and 0.710, respectively. Yan Q et al. (49) used six classifiers (KNN, SVM, XGBoost, RF, LR, and DT) to distinguish NTM-LD from PTB, and the results showed that the LR classifier has the highest precision, recall, and F1-score, which were 0.92, 0.94, and 0.93. Its AUC, sensitivity, and specificity were 0.95, 0.94, 0.87 in the external test. Zhou L et al. (34) compared the discriminatory abilities of four classifiers (XGBoost, LR, SVM, and RF) for NTM-LD and PTB. The results showed that the AUCs of these four classifiers were ≥ 0.7759 in both the training and validation cohorts. However, only SVM demonstrated stable performance in effectively identifying these two diseases.

Unlike the previous studies, in order to enhance the performance and stability of the LR, we preprocessed the data with the YeoJohnson transformation, both alone and combined with LDA, before constructing the model. As a result, in testing cohort, compared with the LR without preprocessing, the AUC, sensitivity, and accuracy of the YeoJohnson_LR model were improved. Moreover, the AUC, sensitivity, specificity, accuracy, and F1 score of YeoJohnson_LR (LDA) were significantly enhanced. Compared with YeoJohnson_LR, YeoJohnson_LR (LDA) demonstrated significant improvements in AUC, specificity, accuracy, precision, and F1 score in the testing cohort. This proves that by applying the YeoJohnson and LDA preprocessing methods before LR modeling, the performance of the model can be further optimized and enhanced. Furthermore, significant reclassification improvement was confirmed between the YeoJohnson_LR and YeoJohnson_LR (LDA) by Delong test (*p* = 0.0094), NRI (0.1703, *p* = 0.0129) and IDI (0.0851, *p* = 0.0003). It indicates that the LDA algorithm holds significant value in improving the application of model reclassification. In addition, the results of the decision curve analysis indicated that the YeoJohnson_LR model yielded a superior net benefit across a wide range of threshold probabilities compared to other models. We also observed that, compared with the PTB group, the radscore values were higher in the NTM-LD group. This suggests that a higher radscore might be more indicative of NTM-LD rather than PTB. However, the radscore calculation may vary depending on the number and type of imaging features included. Therefore, whether there is a critical threshold for distinguishing between NTM-LD and PTB still requires further investigation. Finally, in this study, we compared the YeoJohnson_LR (LDA) model with radiologists. The results showed that our model had higher sensitivity (0.6875) and specificity (0.8904) compared to radiologists. Our model achieved a gain of 3.12–15.62% in sensitivity and 6.85–12.33% in specificity compared to radiologists. This indicates that our model has a lower false negative rate and false positive rate, and has good clinical applicability.

In our study, the three most influential features incorporated into the model, as indicated by their Lasso coefficients, were glszm_wavelet.LLL.ZP (0.104), gldm_log.sigma.2.0.mm.3D.DV (0.059), and glszm_log.sigma.1.0.mm.3D.GLV (0.058). The sign of the Lasso coefficient indicates the direction of its effect on identifying NTM-LD, while its magnitude reflects the strength of influence.

Specifically, ZP quantifies texture coarseness as the ratio of the number of zones to the number of voxels in the ROI; a higher value implies that the ROI is comprised of a greater number of small zones, which signifies a finer, more homogeneous texture. In our model, ZP showed a positive association with NTM-LD (Lasso coefficient = +0.104), suggesting that lesions exhibiting such a finer, more homogeneous texture patterns are more likely to be classified as NTM-LD.

Dependence Variance (DV) DV Measures the variance in dependence size in the image. A higher DV value indicates greater dispersion in dependency sizes, revealing a high degree of spatial irregularity and heterogeneity in local structures. Its positive coefficient (0.059) suggests that imaging patterns with highly disordered and heterogeneous local textures correlate with the diagnosis of NTM-LD.

Gray-Level Variance (GLV): GLV measures the variance in gray level intensities for the zones. A higher GLV value signifies substantial intra-regional density variation, characterized by pronounced fluctuations in pixel values ranging from very low to very high density, thereby indicating strong overall heterogeneity. Its positive coefficient (0.058) suggests that this signature of high overall textural heterogeneity serves as another important criterion for the model in identifying NTM-LD. In summary, our model identifies the feature combination of “high ZP, high DV, and high GLV” as a key indicator for differentiating NTM-LD. This combination collectively defines an imaging texture pattern characterized by “High fineness and homogeneity, high dependence size heterogeneity, and high density heterogeneity” at both local and global scales. While these key features quantify a complex textural phenotype, whether it reflects a single specific change or results from a confluence of multiple imaging findings in NTM-LD cannot be definitively concluded from our current dataset. This is an important question that our future research will aim to address.

According to the literature, there are also some studies that have achieved good results by using different methods. For instance, Hu Y et al. (47) developed A multi-lesion radiomic (MLR) model to distinguish NTM-LD from PTB. The AUC of this model on the internal testing cohort was 90.1% (95% CI, 87.7–92.4%). Li HL et al. (30) constructed a multimodal model incorporating age, IL-6, and the 2 radiomics features, and the optimal model was from the LightGBM algorithm. Its favorable performance was verified in the external test dataset, with an AUC of 0.858, an accuracy of 0.745, and a sensitivity of 0.900. Liu Q et al. (28) established a nomogram prediction model for differentiating NTM-PD from PTB, incorporating six features (TB-IGRA, bronchiectasis, lymph node calcification, pleural effusion, hilar and mediastinal lymph node enlargement) with significant diagnostic value. Its AUC, sensitivity, specificity, and Youden index obtained were 0.938, 0.835, 0.911, and 0.746, respectively. Similarly, Zhang W et al. (29) analyzed the critical differential characteristics between NTM-PD and PTB, and developed a nomogram prediction model based on age, BMI, bronchiectasis, and lung cavitation, which effectively assesses the risk of NTM-PD occurrence. Its AUC, sensitivity, specificity, and accuracy were 0.861, 0.880, 0.740, and 0.833, respectively. Wang L et al. (15) developed a deep learning framework (3D-ResNet) based on CT images to distinguish NTM-PD from PTB. As a result, the AUC in the external test set was 0.78, the sensitivity was 0.75, and the specificity was 0.63. Park M et al. (51) constructed an ensemble model (ResNet 50 + EfficientNet B4) based on chest X-ray images and also achieved good results. The AUC, precision, and accuracy of this model for diagnosing NTM-LD on the external test set were 0.80, 0.82, and 0.78, respectively. Ying C et al. (14) combined T-SPOT with a deep learning model and discovered that it can greatly improve the classification precision of NTM-PD and PTB when the two methods of prediction are consistent. Therefore, it can be seen that the machine model has certain value in distinguishing NTM-LD from PTB, but there are differences in diagnostic performance among different models. To assist clinical diagnosis, developing a robust model that can adapt to multiple centers and devices will be a key issue to be addressed in the future.

This study is meaningful. First, current diagnosis of NTM-LD and PTB mainly relies on bacterial culture and species identification. Our model provides a rapid and cost-effective auxiliary diagnostic approach. It operates using chest CT images, making it easy to adopt across healthcare institutions at all levels, especially in primary care settings with limited resources. It is expected to shorten the diagnostic cycle, reduce medical costs, and provide immediate reference for clinical decision-making. Second, traditional imaging diagnosis depends on radiologists’ subjective visual assessment, which is easily influenced by experience. Our model analyzes CT images by quantifying imaging indicators, offering objectivity. This reduces interference from human subjectivity and enhances the standardization and reproducibility of the diagnostic process. Third, the study involved 1,512 cases from two centers, which supplements the shortcomings of small sample size and single center in previous studies on radiomics machine learning (28, 29, 47, 50).

Finally, previous studies focused on comparing the diagnostic performance of various classifiers in machine learning (30, 34). Based on the previous research, we added a new data preprocessing method, which was an innovative attempt and achieved satisfactory results.

However, this study also has some limitations. Firstly, this study only distinguishes between NTM-LD and PTB. In addition, NTM-LD needs to be differentiated from various other diseases, such as viral or fungal infections, tumors, and so on, which will be even more challenging in actual clinical scenarios. Future studies will incorporate a broader range of diseases to explore the model’s ability to identify diseases across a wider spectrum. Secondly, the model is constructed based on radiomics features and does not incorporate clinical characteristics or laboratory results. Future studies will incorporate more types of data to build the model and explore more information with potential diagnostic value.

## Conclusion

In conclusion, this study developed a radiomics machine learning YeoJohnson_LR (LDA) model based on CT images for differentiating NTM-LD from PTB. Compared with radiologists, this model has higher diagnostic efficacy and may potentially become a screening tool for patients with pulmonary mycobacteria. Compared with the time-consuming bacterial culture, the acquisition of CT images is instantaneous. Our model can assist in the early diagnosis when clinicians suspect that patients have NTM-LD or PTB, thereby benefiting the patients.

## Data Availability

The original contributions presented in the study are included in the article/Supplementary material, further inquiries can be directed to the corresponding author.

## Glossary

AFB: Acid-fast bacilli
AUC: area under the curve
BMI: body mass index
CI: confidence interval
CT: computed tomography
DSC: Dice similarity coefficient
DT: Decision tree
DV: DependenceVariance
GLCM: gray level co-occurrence matrix
GLDM: gray level dependence matrix
GLRLM: gray level run length matrix
GLSZM: gray level size zone matrix
GLV: GrayLevelVariance
IDI: integrated discrimination improvement
IGRA: interferon-*γ* release assay
KNN: k-Nearest Neighbor
LASSO: Least Absolute Shrinkage and Selection Operator
LR: Logistic Regression
LTBI: latent tuberculosis infection
ML: machine learning
NGTDM: neighboring gray tone difference matrix
NRI: net reclassification index
NTM: nontuberculous mycobacteria
NTM-LD: non-tuberculous mycobacterial lung disease
PTB: pulmonary tuberculosis
QDA: Quadratic Discriminant Analysis
RF: Random Forest
ROC: receiver operating characteristic
SVM: Support Vector Machin
XGBoost: eXtreme Gradient Boosting
ZP: ZonePercentage
