# Supplementary material for: Distinguishing nontuberculous mycobacterial lung disease from pulmonary tuberculosis using radiomics machine learning models from CT images
Source: Front Med (Lausanne). 2026 Jan 21;13:1721949. doi: 10.3389/fmed.2026.1721949 (PMC12868291; doi:10.3389/fmed.2026.1721949)
Supplement: Supplementary file 1 [file Supplementary_file_1.docx]

**Supplementary Material 1: Step-by-step workflow of the radiomics model development and validation**

1. **Cohort Establishment**: Patients with microbiologically confirmed PTB or NTM-LD were enrolled from two centers according to explicit inclusion/exclusion criteria. The cohort was then split into a training/validation set (Center 1) and an independent testing set (Center 2).
2. **CT Standardization & Lung Segmentation**: All CT images were interpolated to a uniform voxel size. Bilateral lungs were automatically segmented by a deep learning model, followed by mandatory expert review and manual correction.
3. **Radiomics Feature Extraction & Normalization**: 2,264 radiomics features were extracted from the segmented volumes. Two parallel normalization strategies (Z-score and Yeo-Johnson transformation) were applied, creating two feature sets for subsequent analysis.
4. **Feature Selection**: For each normalized feature set, a two-step selection was performed: (a) univariate filtering (correlation analysis, *p* < 0.05), followed by (b) LASSO regression with 10-fold cross-validation to identify the most predictive non-redundant features. A Radscore was calculated for each patient.
5. **Model Construction & Selection**: Using the selected features, multiple models were built on the training set using Logistic Regression (LR), Random Forest (RF), and Quadratic Discriminant Analysis (QDA) classifiers, exploring combinations with/without Linear Discriminant Analysis (LDA) dimensionality reduction. The optimal hyperparameters and model type were determined using the validation set.
6. **Comprehensive Evaluation**: The final chosen model was locked and evaluated on the independent test set from Center 2. Its performance was rigorously assessed via ROC analysis, calibration, and DCA. Its discriminative ability was statistically compared against other models and benchmarked against the independent assessments of three blinded radiologists.
