# Supplementary material for: Distinguishing nontuberculous mycobacterial lung disease from pulmonary tuberculosis using radiomics machine learning models from CT images
Source: Front Med (Lausanne). 2026 Jan 21;13:1721949. doi: 10.3389/fmed.2026.1721949 (PMC12868291; doi:10.3389/fmed.2026.1721949)
Supplement: Supplementary file 2 [file Supplementary_file_2.docx]

# Supplementary materials 2

**Supplementary Material 2: Radiomics features**

**Supplementary table 1: feature selection result**

| Radiomics features | Lasso coefficient |
| --- | --- |
| wavelet_glszm_wavelet.LLL.ZonePercentage | 0.104055822 |
| log_gldm_log.sigma.2.0.mm.3D.DependenceVariance | 0.0588175 |
| log_glszm_log.sigma.1.0.mm.3D.GrayLevelVariance | 0.057955567 |
| log_glszm_log.sigma.2.0.mm.3D.LargeAreaHighGrayLevelEmphasis | 0.052028798 |
| log_gldm_log.sigma.1.0.mm.3D.DependenceVariance | 0.050151456 |
| wavelet_glcm_wavelet.LHH.ClusterShade | 0.042105198 |
| mean_gldm_LargeDependenceHighGrayLevelEmphasis | 0.035670362 |
| recursivegaussian_glrlm_ShortRunLowGrayLevelEmphasis | 0.035132 |
| wavelet_glcm_wavelet.HLL.Idmn | 0.03272596 |
| curvatureflow_glrlm_ShortRunEmphasis | 0.031374626 |
| log_gldm_log.sigma.2.0.mm.3D.DependenceNonUniformityNormalized | 0.02929793 |
| wavelet_firstorder_wavelet.LHH.Skewness | 0.022642506 |
| original_shape_Elongation | 0.021383146 |
| boxsigmaimage_firstorder_Maximum | 0.018426752 |
| boxsigmaimage_glszm_LargeAreaEmphasis | 0.01596312 |
| laplaciansharpening_firstorder_Range | 0.015809773 |
| wavelet_glcm_wavelet.LHL.Imc1 | 0.013549466 |
| wavelet_firstorder_wavelet.HHL.Uniformity | 0.012881495 |
| log_glszm_log.sigma.2.0.mm.3D.HighGrayLevelZoneEmphasis | 0.012676399 |
| normalize_firstorder_InterquartileRange | 0.012068785 |
| log_firstorder_log.sigma.1.0.mm.3D.Kurtosis | 0.010394632 |
| laplaciansharpening_glrlm_ShortRunHighGrayLevelEmphasis | 0.010225241 |
| log_firstorder_log.sigma.4.0.mm.3D.Skewness | 0.008845481 |
| wavelet_glszm_wavelet.HHH.SmallAreaEmphasis | 0.008521311 |
| specklenoise_glcm_Imc2 | 0.007644118 |
| normalize_glrlm_RunLengthNonUniformityNormalized | 0.006975282 |
| wavelet_glcm_wavelet.HLH.ClusterShade | 0.006722236 |
| log_ngtdm_log.sigma.4.0.mm.3D.Contrast | 0.006650851 |
| additivegaussiannoise_ngtdm_Strength | 0.006165361 |
| wavelet_glszm_wavelet.HHL.GrayLevelNonUniformityNormalized | 0.006146107 |
| normalize_glszm_SmallAreaLowGrayLevelEmphasis | 0.005571402 |
| original_ngtdm_Strength | 0.004504013 |
| shotnoise_ngtdm_Strength | 0.004384591 |
| wavelet_glszm_wavelet.LHL.LargeAreaHighGrayLevelEmphasis | 0.002525968 |
| wavelet_glrlm_wavelet.LHL.LongRunHighGrayLevelEmphasis | 0.001155598 |
| normalize_gldm_LargeDependenceHighGrayLevelEmphasis | 0.000691053 |
| laplaciansharpening_glcm_Id | -2.35E-17 |
| wavelet_firstorder_wavelet.HHL.Energy | -7.25E-09 |
| recursivegaussian_glrlm_RunLengthNonUniformity | -0.000316321 |
| wavelet_glszm_wavelet.HLL.GrayLevelNonUniformity | -0.000753054 |
| log_ngtdm_log.sigma.0.5.mm.3D.Complexity | -0.000804937 |
| discretegaussian_glrlm_RunLengthNonUniformity | -0.001068106 |
| mean_ngtdm_Busyness | -0.001211599 |
| wavelet_gldm_wavelet.HLL.SmallDependenceLowGrayLevelEmphasis | -0.001279604 |
| wavelet_glcm_wavelet.HLL.InverseVariance | -0.001715856 |
| log_gldm_log.sigma.4.0.mm.3D.SmallDependenceLowGrayLevelEmphasis | -0.001869013 |
| mean_glszm_ZonePercentage | -0.002712487 |
| specklenoise_ngtdm_Contrast | -0.003100154 |
| log_glszm_log.sigma.0.5.mm.3D.SmallAreaHighGrayLevelEmphasis | -0.00311224 |
| wavelet_gldm_wavelet.LHH.SmallDependenceLowGrayLevelEmphasis | -0.003167392 |
| curvatureflow_glrlm_RunLengthNonUniformity | -0.003497689 |
| specklenoise_gldm_GrayLevelVariance | -0.0039753 |
| original_gldm_SmallDependenceLowGrayLevelEmphasis | -0.004450462 |
| specklenoise_firstorder_Minimum | -0.004575273 |
| wavelet_glcm_wavelet.HLL.ClusterShade | -0.006131856 |
| wavelet_glszm_wavelet.LHH.GrayLevelNonUniformity | -0.006197692 |
| shotnoise_glrlm_RunLengthNonUniformity | -0.006710638 |
| binomialblurimage_glszm_SizeZoneNonUniformityNormalized | -0.006946493 |
| wavelet_glcm_wavelet.LLL.Correlation | -0.008797571 |
| laplaciansharpening_glcm_Idm | -0.00888105 |
| log_firstorder_log.sigma.4.0.mm.3D.RootMeanSquared | -0.009439697 |
| specklenoise_firstorder_Maximum | -0.009677714 |
| binomialblurimage_glrlm_LongRunLowGrayLevelEmphasis | -0.010757619 |
| laplaciansharpening_glcm_Idn | -0.011293139 |
| wavelet_glcm_wavelet.LLL.Idmn | -0.011857134 |
| wavelet_ngtdm_wavelet.LLL.Complexity | -0.012191961 |
| binomialblurimage_glszm_GrayLevelNonUniformity | -0.01332921 |
| additivegaussiannoise_glrlm_LongRunHighGrayLevelEmphasis | -0.013661621 |
| wavelet_glszm_wavelet.LHL.LargeAreaLowGrayLevelEmphasis | -0.015556329 |
| shotnoise_glszm_SmallAreaHighGrayLevelEmphasis | -0.016662246 |
| normalize_glszm_ZonePercentage | -0.018100599 |
| normalize_gldm_SmallDependenceLowGrayLevelEmphasis | -0.022611693 |
| wavelet_glszm_wavelet.HHH.GrayLevelNonUniformityNormalized | -0.024847496 |
| original_glcm_ClusterShade | -0.02894252 |
| log_firstorder_log.sigma.1.0.mm.3D.Range | -0.030836906 |
| wavelet_firstorder_wavelet.HHL.TotalEnergy | -0.034868125 |
| log_glrlm_log.sigma.4.0.mm.3D.GrayLevelVariance | -0.03688513 |
| recursivegaussian_glcm_Imc1 | -0.03920412 |
| log_glszm_log.sigma.1.0.mm.3D.ZonePercentage | -0.04075324 |
| log_gldm_log.sigma.4.0.mm.3D.DependenceVariance | -0.045922555 |
| wavelet_gldm_wavelet.HHL.DependenceEntropy | -0.048516166 |
| boxsigmaimage_glcm_Imc1 | -0.048575264 |
| binomialblurimage_glcm_Correlation | -0.053338483 |
| binomialblurimage_glrlm_RunEntropy | -0.06380225 |
| log_glcm_log.sigma.4.0.mm.3D.ClusterProminence | -0.06477872 |
|  |  |

**Supplementary Table 2. The top 15 discriminative features demonstrating significant differences between**

**NTM-LD and PTB groups**

| Features | NTM-LD | | PTB | difference | *p* value | effect size | adjusted *p* value |
| --- | --- | --- | --- | --- | --- | --- | --- |
| binomialblurimage_glcm_Correlation | 0.711 | 0.667 | | 0.044 | <0.001 | 0.569 | <0.001 |
| wavelet_glcm_wavelet.LLL.Correlation | 0.729 | 0.693 | | 0.036 | <0.001 | 0.500 | <0.001 |
| original_glcm_ClusterShade | 40.308 | 31.857 | | 8.451 | <0.001 | 0.427 | <0.001 |
| log_firstorder_log.sigma.1.0.mm.3D.Kurtosis | 7.648 | 8.211 | | 0.564 | <0.001 | -0.451 | <0.001 |
| normalize_firstorder_InterquartileRange | 0.398 | 0.311 | | 0.087 | <0.001 | 0.378 | <0.001 |
| specklenoise_firstorder_Maximum | 2233.385 | 2132.859 | | 100.526 | <0.001 | 0.369 | <0.001 |
| original_shape_Elongation | 0.785 | 0.815 | | 0.031 | <0.001 | -0.363 | <0.001 |
| log_firstorder_log.sigma.4.0.mm.3D.Skewness | 0.122 | 0.185 | | 0.063 | <0.001 | -0.361 | <0.001 |
| wavelet_glszm_wavelet.LHH.GrayLevelNonUniformity | 6045.416 | 5297.833 | | 747.582 | <0.001 | 0.333 | <0.001 |
| binomialblurimage_glrlm_RunEntropy | 4.451 | 4.406 | | 0.045 | <0.001 | 0.333 | <0.001 |
| log_glcm_log.sigma.4.0.mm.3D.ClusterProminence | 19.183 | 17.825 | | 1.358 | <0.001 | 0.315 | <0.001 |
| normalize_glrlm_RunLengthNonUniformityNormalized | 0.121 | 0.126 | | 0.005 | <0.001 | -0.330 | <0.001 |
| wavelet_glcm_wavelet.LLL.Idmn | 0.993 | 0.993 | | 0.000 | <0.001 | -0.317 | <0.001 |
| normalize_gldm_LargeDependenceHighGrayLevelEmphasis | 689.960 | 673.390 | | 16.571 | <0.001 | 0.296 | <0.001 |
| log_gldm_log.sigma.4.0.mm.3D.DependenceVariance | 42.568 | 42.212 | | 0.356 | <0.001 | 0.314 | <0.001 |
